# Combination of Immunotherapy and Radiotherapy for Recurrent Malignant Gliomas: Results From a Prospective Study

**DOI:** 10.3389/fimmu.2021.632547

**Published:** 2021-05-07

**Authors:** Haihui Jiang, Kefu Yu, Yong Cui, Xiaohui Ren, Mingxiao Li, Chuanwei Yang, Xuzhe Zhao, Qinghui Zhu, Song Lin

**Affiliations:** ^1^ Department of Neurosurgery, Beijing Tiantan Hospital, Capital Medical University, Beijing, China; ^2^ National Clinical Research Center for Neurological Diseases, Center of Brain Tumor, Beijing Institute for Brain Disorders and Beijing Key Laboratory of Brain Tumor, Beijing, China; ^3^ Department of Pharmacy, Beijing Tiantan Hospital, Capital Medical University, Beijing, China; ^4^ Beijing Neurosurgical Institute, Capital Medical University, Beijing, China

**Keywords:** malignant gliomas, immunotherapy, reirradiation, immunoadjuvant, immuno-oncology

## Abstract

**Background:**

World Health Organization (WHO) grade IV glioma remains one of the most lethal tumors with a dismal prognosis and inevitable recurrence. We evaluated the safety and efficacy of immunotherapy with radiotherapy in this population of patients.

**Methods:**

This study was a single-arm, open-label, phase I trial based on patients with recurrent WHO grade IV glioma. Patients were treated with intracranial and systemic immunoadjuvants in combination with low-dose reirradiation. The primary endpoint of the present trial was safety. Secondary endpoints were overall survival (OS) and progression-free survival (PFS). This trial is registered at ClinicalTrials.gov, NCT03392545.

**Results:**

Thirty patients were enrolled. The most common adverse events (AEs) were fever (66.7%), vomiting (33.3%), headache (30.0%), and fatigue (23.3%). Only a single patient experienced grade 3 fever, and no grade 4 AEs or deaths related to treatment were observed. Of the 30 patients, 1 (3.3%) had a complete response, 5 (16.7%) had a partial response, 9 (30.0%) had stable disease, and 15 (50.0%) had progressive disease, resulting in an objective response rate of 20.0%. The median PFS of the entire cohort was 88.0 (61.0-254.0) days, and the median OS was 362.0 (197.0-601.0) days. Patients could be divided into responders and non-responders, and these groups exhibited a significant difference in terms of survival time, T lymphocyte subsets, frequency of cell division cycle 27 (CDC27) mutation status, and CD15 and CD68 expression (*P*<0.05).

**Conclusion:**

The combination of immunotherapy and radiotherapy is well tolerated and may provide clinical benefit for patients with recurrent WHO grade IV glioma. A prospective phase II study is needed to further validate the efficacy of our therapeutic regimen.

## Introduction

World Health Organization (WHO) grade IV malignant glioma, including glioblastoma (GBM) with wild-type or mutant isocitrate dehydrogenase (IDH) and diffuse midline glioma (DMG) with H3K27M mutation, is the most common primary central nervous system tumor and confers a poor prognosis (1). The current treatment for patients with GBM involves maximal safe resection, radiotherapy, temozolomide (TMZ) based chemotherapy, and even the latest tumor treating field (TTF) therapy (2–4). Despite these multimodal approaches, the median survival of GBM is still less than 24 months and relapse after therapy is inevitable (2, 5). During recurrence, treatment options are less well defined and no interventions have shown encouraging efficacy (6). Hence, there is an urgent need for more effective therapies for recurrent WHO grade IV gliomas.

Immuno-oncology, which has prominently transformed the management of many cancers, has indicated that immunotherapy is the most promising treatment for grade IV gliomas (7–10). Since the discovery of central nervous system lymphatic vessels (11), immunotherapies, including immune checkpoint inhibitors (12), chimeric antigen receptor (CAR) T cell therapy (13), vaccines (14), and oncolytic virus (15), have been attempted to treat malignant gliomas. However, due to some challenging factors (16), such as the existence of intact blood-brain barrier (BBB) of tumor region, intratumor heterogeneity, and the unique immunosuppressive microenvironment, no significant benefit of these regimens has been observed in clinical practice (6, 17).

Recently, radiotherapy concurrent with immunotherapy has made great strides in the treatment of various tumors (18–20). It has been reported that radiotherapy in combination with immunotherapy can induce a synergistic effect *via* immunomodulation (18, 21). Radiotherapy can enhance the immunologic response to tumors by creating an *in situ* vaccine by eliciting antigen released from dying tumor cells (22, 23). In this study, to obtain more favorable antitumor activity, radiotherapy was delivered in conjunction with intracranial and systemic immunoadjuvants, a combination which has been shown to strengthen the efficacy of tumor antigen vaccination (24). Therefore, the present trial was designed to evaluate the safety and immunological efficacy of low-dose reirradiation in combination with polyinosinic:polycytidylic acid (poly I:C) and granulocyte-macrophage colony stimulating factor (GM-CSF) in adult patients with recurrent WHO grade IV glioma.

## Materials and Methods

### Study Design and Participants

This study was a single-arm, open-label, phase I trial in patients with recurrent WHO grade IV glioma. Patients were enrolled in Beijing Tiantan Hospital, an affiliate of Capital Medical University, on the basis of the following inclusion criteria: aged 18-65 years, pathologically confirmed recurrent WHO grade IV glioma by resection or biopsy, amount of corticosteroids was no more than 2 mg/day, Karnofsky performance scale (KPS) score of 70 or higher, and adequate hematological, hepatic, renal, and coagulation function. Exclusion criteria included previous medical treatment for other malignancies, systemic inflammatory and immune system diseases, allergy to immunoadjuvants, and pregnancy or lactation.

### Procedures

**Figure 1:**
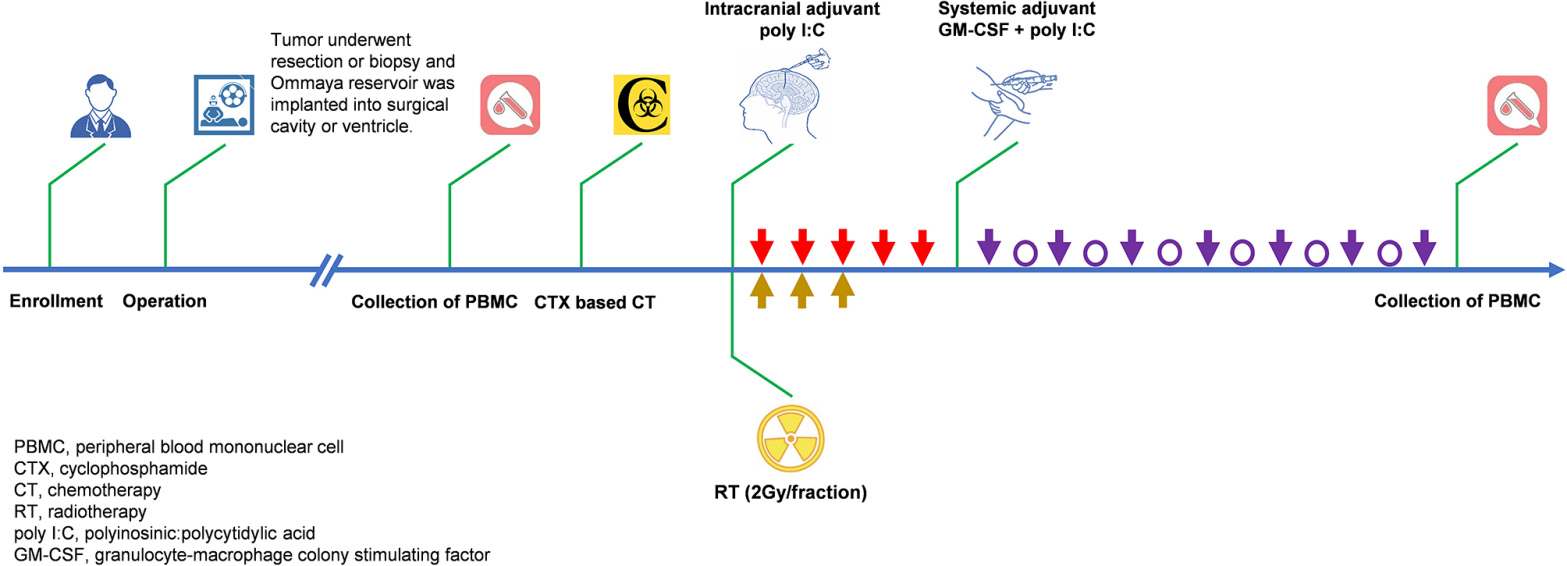
Treatment scheme for patients enrolled in the present study.

The study procedures are elaborated in detail in **Figure 1**. Patients received low-dose cyclophosphamide (CTX) intravenously 24 hours before immunoadjuvant treatment to eliminate regulatory T cells (25, 26). Then, intracranial and systemic adjuvants were successively administered. Intracranial immunoadjuvant was poly I:C, which was infused into a surgical cavity or ventricle with a dose of 1-2 mg per shot, qd, for a total of 5 shots, with the first three shots concomitant to radiation (2.0 Gy/fraction). Systemic immunoadjuvants consisted of poly I:C (50 μg/kg per shot, qod, 7 shots, intramuscular) and GM-CSF (125μg/m^2^ per shot, qod, 7 shots, subcutaneous). This treatment continued until disease progression or onset of intolerable toxic effects. Patients could restart this treatment after the first evidence of progression, until confirmed by follow-up magnetic resonance imaging (MRI) within 12 weeks if there was evidence of clinical activity and adequate tolerability. Tumor assessments were performed with contrast-enhanced MRI at an interval of 8 weeks. Treatment response was defined as complete response (CR), partial response (PR), stable disease (SD), or progressive disease (PD) by the investigators based on immunotherapy response assessment in neuro-oncology (iRANO) criteria (27). Adverse events (AEs) were evaluated according to the National Cancer Institute (NCI) Common Terminology Criteria for Adverse Events (CTCAE) (version 4.0). Immunological response was also assessed one day prior to CTX-based chemotherapy and one day post the last shot of systemic immunoadjuvant by flow cytometry assays on peripheral blood mononuclear cells (PBMCs).

### Outcomes

The primary endpoint of this study was safety. Patients were monitored continuously for AEs at each clinic visit and AEs were graded according to CTCAE Version 4.0. Secondary endpoints included progression-free survival (PFS) and overall survival (OS). OS was defined as the time from the date of CTX-based chemotherapy to the date of death or last follow-up. PFS was defined as the time from the date of CTX-based chemotherapy to the date of progression or last follow-up.

### Flow Cytometry Assay

Patient blood samples were collected by venipuncture. All peripheral blood samples (5 ml per subject) were collected in vacutainer tubes (BD Biosciences, San Jose, CA, USA) containing ethylenediaminetetraacetic acid (EDTA). For surface staining, 100 µL of heparinized peripheral blood was added to tubes containing 10 µL of mouse anti-human monoclonal antibodies (mAbs), including Peridinin Chlorophyll Protein Complex (PerCP)-conjugated anti-CD45, FITC-conjugated anti-CD3, APC-conjugated anti-CD4, PE-conjugated anti-CD8, and PE-conjugated anti-CD16+CD56, which were provided by ACEA Biosciences (San Diego, CA, USA). Isotype-matched mouse anti-human IgG antibodies served as negative controls for all fluorescein-conjugated IgG mAbs. Thereafter, the blood was mixed with the cocktail monoclonal antibody solution and incubated for 15 min at room temperature. Then, a lysing solution (OptiLyse C, Beckman Coulter) was added, and the mixture was incubated for another 15 min. To detect the percentage and absolute cell numbers of different subsets in peripheral blood, cells were collected and analyzed on a NovoCyte Flow Cytometer (ACEA Biosciences, San Diego, CA, USA) using NovoExpress Software (ACEA Biosciences, San Diego, CA, USA).

### Whole Exome Sequencing (WES)

Tumor and matched blood samples from patients were collected. Then DNA was extracted using Qiagen DNeasy Blood & Tissue Kit (#69504), and quantified by means of Nanodrop ND-100 (Thermo Scientific, Waltham, MA) and Qubit 2.0 Fluorometer (Life Technologies, Carlsbad, CA, USA). DNA was captured and amplified with Agilent Technologies SureSelect Human All Exon version 5 (Agilent Technologies, Santa Clara, CA, USA), followed by paired-end sequencing (2 × 125 cycles) on a HiSeq2500 platform (Illumina Inc., San Diego, CA, USA), according to the manufacturer’s protocol. Raw image analysis and base calling were performed using the Illumina onboard RTA3 program with default parameters. After removing adapters and low-quality reads, the remaining reads were aligned to NCBI human genome reference assembly hg19 using the Burrows-Wheeler Aligner (BWA) tool and further processed using the Genome Analysis Toolkit (GATK, version 3.5), including the GATK Realigner Target Creator to identify regions that needed to be realigned. Single-nucleotide variants (SNVs), Indels, and copy number variation (CNV) were assessed using ANNOVAR, VarscanIndel, and CNVnator software, respectively (28). During mutation calling, the reads from the tumor sample were compared with those from the paired blood from the same patient to generate a list of somatic mutations. The called somatic mutations were then filtered and annotated using the Variant Effect Predictor (VEP) package (hg19 version) (29).

### Imaging Mass Cytometry (IMC)

Metal-labeled antibodies were prepared according to the Fluidigm protocol. The antibody panel included lymphocyte types, cytokine expression, lymphocyte activation, vascular and spatial structure of cells from other tissues. Metal-conjugated primary antibodies were prepared with a Maxpar labeling kit (Fluidigm). The antibodies were diluted in antibody stabilization solution (Candor Bioscience GmbH, Wangen, Germany) for long-term storage at 4°C. Descriptions of the antibodies, isotope tags, clones, and concentrations used for staining are shown in **Table S1**.

Tumor samples were fixed in formalin and embedded in paraffin. Sections with a thickness of 5 µm were baked at 60°C for 2 hours, deparaffinized in xylene, and hydrated in a graded series ethanol (100%, 95%, 80%, 70%) for 5 min each. Next, 40 mL Antigen Retrieval Reagent-Basic (R&D Systems, diluted from 10× to 1×) was added to conical tubes, and the tubes were further incubated on a heating block (97°C) with loose lids. After immediate cooling to 60°C for 20 min, the sections were then blocked with 3% bovine serum albumin (BSA) for 45 min at room temperature. For staining, the sections were incubated overnight at 4°C with an antibody master mix. Samples were washed twice in 0.1% Triton X-100 in PBS for 8 min with slow agitation in Coplin jars. Sections were then stained with Intercalator-Ir (Fluidigm; cat. no. 201192A) in PBS for 30 min at room temperature. Slides were air dried and stored at 4°C for ablation.

According to hematoxylin-eosin staining, we selected the appropriate 500 × 500 µm location for laser-based cell ablation and imaging. IMC images were acquired using a Hyperion Imaging System (Fluidigm). The largest square area was laser-ablated in a rastered pattern at 200 Hz, and preprocessing of the raw data was completed with commercial acquisition software (Fluidigm). IMC acquisition stability was monitored by interspersed acquisition of an isotope-containing polymer (Fluidigm). All successful image acquisitions were processed using MCDViewer, CellProfilor, and HistoCAT. R scripts were used to quantify cell number, generate t-distributed stochastic neighbor embedding (t-SNE) plots and perform neighborhood analysis.

### Statistical Analysis

Patients who experienced CR, PR, or SD were regarded as responders, while those with PD were regarded as non-responders. Categorical comparisons between responders and non-responders were performed using the chi-square test or Fisher’s exact test, as appropriate. Differences in age at diagnosis were evaluated by Student’s t-test. The survival rate was estimated using the Kaplan-Meier method, and differences between subgroups were compared by the log-rank test. For the assessment of immunological response after receiving intracranial and systemic immunoadjuvants, paired t tests were used to compare the numbers of CD4^+^ T, and CD8^+^ T cells and natural killer (NK) cells before and after the immunoadjuvant treatments. All tests were two-sided, and a P value less than 0.05 was considered to indicate statistical significance. All analyses were performed with SPSS Statistics software for Windows version 26.0 (IBM Corporation, Armonk, NY, USA) and R software (https://cran.r-project.org).

## Results

### Demographics and Clinical Characteristics

Between January 2018 and December 2019, thirty patients with a diagnosis of recurrent WHO grade IV glioma were enrolled in the present study. Of these patients, there were 21 males and 9 females, and the mean age was 43.0 ± 13.2 (range, 18-65) years. The baseline characteristics are shown in **Table 1**. Fourteen (46.7%) patients had tumors located in a single lobe. After chemoradiotherapy, 10 (33.3%) patients experienced local recurrence and 20 (66.7%) patients experienced distant recurrence (**Figure S1**). All diagnoses of tumor recurrence were confirmed by operation and histopathology, including 13 (43.3%) surgical resections and 17 (56.7%) biopsies. According to the 2016 WHO classification scheme, there were 19 (63.3%) IDH-wildtype GBM, 6 (20.0%) IDH-mutant GBM, and 5 (16.7%) H3K27M-mutant DMG. The frequencies of KPS scores of 70, 80, 90, and 100 during recurrence were 33.3%, 30.0%, 23.3%, and 13.3%, respectively. All patients were available for the assessment of O^6^-methylguanine-DNA-methyltransferase (MGMT), and 10 (33.3%) patients were identified to have a methylated MGMT promoter.

**Table 1:** Comparison of clinicopathologic data of responders and non-responders.

Variable	Responder (n = 15)	Non-responder (n = 15)	P value
Age at diagnosis (years)	48.3 ± 10.6	37.8 ± 13.9	**0.028**
Gender (n, %)			0.427^#^
Male	12(80.0%)	9(60.0%)	
Female	3(20.0%)	6(40.0%)	
Tumor location			0.143
Single lobe	9(60.0%)	5(33.3%)	
Multiple lobes	6(40.0%)	10(66.7%)	
Previous chemoradiotherapy			
Yes	15(100.0%)	15(100.0%)	NA
No	0(0.0%)	0(0.0%)	
KPS score			0.672
70	4(26.7%)	6(40.0%)	
80	6(40.0%)	3(20.0%)	
90	3(20.0%)	4(26.7%)	
100	2(13.3%)	2(13.3%)	
Recurrence pattern			**0.020**
Local	8(53.3%)	2(13.3%)	
Distant	7(46.7%)	13(86.7%)	
Extent of resection			0.269
Resection	8(53.3%)	5(33.3%)	
Biopsy	7(46.7%)	10(66.7%)	
Pathology subtypes			0.632
IDH-wildtype GBM	9(60.0%)	10(66.7%)	
IDH-mutant GBM	4(26.7%)	2(13.3%)	
H3K27M-mutant DMG	2(13.3%)	3(20.0%)	
MGMT promoter			0.439
Methylated	6(40.0%)	4(26.7%)	
Unmethylated	9(60.0%)	11(73.3%)	

KPS, Karnofsky performance scale; DMG, diffuse midline glioma; IDH, isocitrate dehydrogenase; GBM, glioblastoma; MGMT, O^6^-methylguanine-DNA-methyltransferase; NA, not applicable.

^#^Fisher exact test. Bold values mean a p value less than 0.05.

### Safety

Patients who received at least one dose of immunoadjuvants and reirradiation were included in the analysis. The treatment-related AEs are summarized in **Table 2**. Overall, the treatment was safe and well tolerated. The most common AEs were flu-like symptoms including fever (66.7%), vomiting (33.3%), headache (30.0%), and fatigue (23.3%). Only a single patient experienced grade 3 fever possibly related to the immunoadjuvants. All these symptoms could be controlled with routine supporting therapies and symptomatic treatments. There were no grade 4 AEs or deaths attributable to this regimen. PD was the most common cause of treatment discontinuation.

**Table 2:** Adverse events of patients enrolled in this study.

Variable	Grade 1	Grade 2	Grade 3	Grade 4
Hematologic toxicity				
Anemia	1(3.3%)	0(0.0%)	0(0.0%)	0(0.0%)
Thrombocytopenia	1(3.3%)	0(0.0%)	0(0.0%)	0(0.0%)
Neutropenia	2(6.7%)	1(3.3%)	0(0.0%)	0(0.0%)
Lymphopenia	1(3.3%)	0(0.0%)	0(0.0%)	0(0.0%)
Nervous system disorder				
Hypersomnia	0(0.0%)	2(6.7%%)	0(0.0%)	0(0.0%)
Seizure	1(3.3%)	1(3.3%)	0(0.0%)	0(0.0%)
Headache	6(20.0%)	3(10.0%)	0(0.0%)	0(0.0%)
Gastrointestinal disorder				
Nausea	2(6.7%)	3(10.0%)	0(0.0%)	0(0.0%)
Vomiting	8(26.7%)	2(6.7%)	0(0.0%)	0(0.0%)
Diarrhea	0(0.0%)	1(3.3%)	0(0.0%)	0(0.0%)
General disorder				
Fever	14(46.7%)	5(16.7%)	1(3.3%)	0(0.0%)
Chills	3(10.0%)	0(0.0%)	0(0.0%)	0(0.0%)
Fatigue	3(10.0%)	4(13.3%)	0(0.0%)	0(0.0%)
Others				
Arthralgia	1(3.3%)	0(0.0%)	0(0.0%)	0(0.0%)
Rash maculo-papular	0(0.0%)	3(10.0%)	0(0.0%)	0(0.0%)

### Clinical Efficacy and Immunological Response

**Figure 2:**
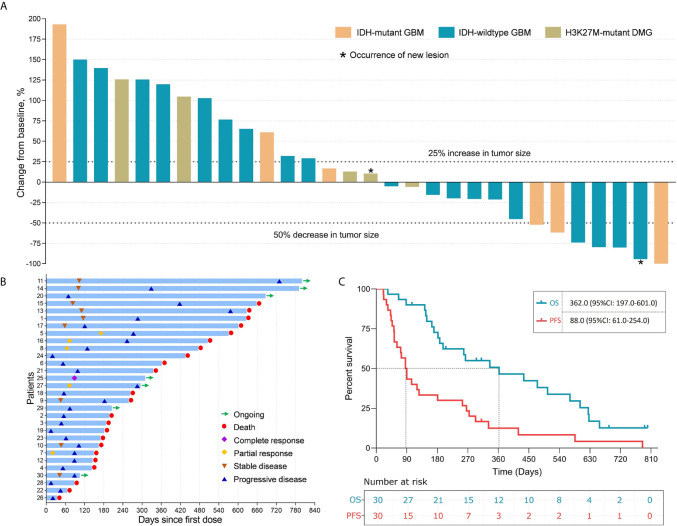
The clinical efficacy of immunoadjuvant treatment and reirradiation in patients with recurrent WHO grade IV gliomas. **(A)** Waterfall plot showing the best tumor response in patients treated with immunoadjuvant therapy and reirradiation. **(B)** Swimmer plot showing disease status and survival time in 30 patients treated with immunoadjuvant therapy and reirradiation. **(C)** The median progression-free survival and overall survival of patients treated with immunoadjuvant therapy and reirradiation.

Among the 30 patients, 1 (3.3%, patient 25) experienced CR and 5 (16.7%) experienced PR, which contributed to an objective response rate (ORR) of 20.0%. Nine (30.0%) patients had SD for 40 days to 118 days after the first infusion of intracranial immunoadjuvant. Fifteen (50.0%) patients experienced PD with a median time to progression of 52.0 (95% confidence interval [CI]: 43.5-60.5) days. The time-on-study for all enrolled patients is shown in **Figures 2A, B**. Notably, patient 2 and patient 22 showed radiological responses that were categorized as PD because of the occurrence of new lesions (**Figures S2**, **S3**). At a median follow-up of 693.0 days, a total of 29 (96.7%) patients progressed, and 23 (76.7%) patients died. The median PFS of the entire cohort was 88.0 (95% CI: 61.0-254.0) days, and the median OS was 362.0 (95% CI: 197.0-601.0) days (**Figure 2C**).

**Figure 3:**
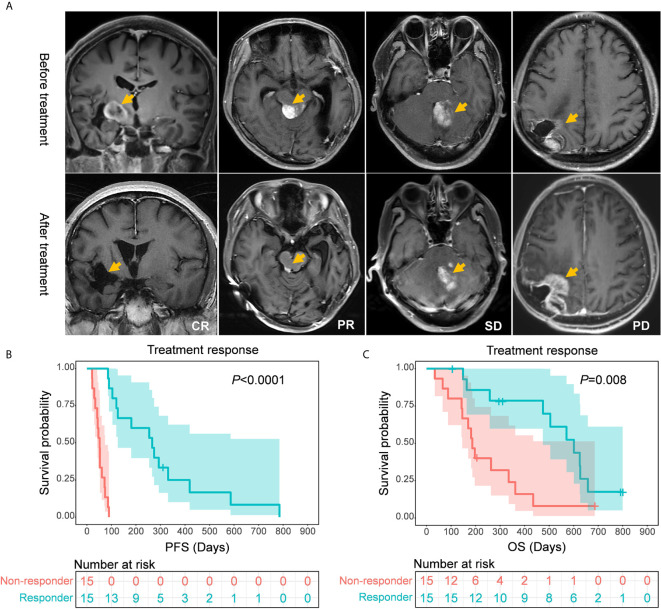
**(A)** Representative MR images of patients with different treatment responses. **(B, C)** Comparisons of survival rates between responders and non-responders. Responders showed a significantly longer progression-free survival (*P* < 0.0001) and overall survival (*P*=0.008) than non-responders.

On the basis of treatment response, patients who had CR, PR, or SD were defined as responders in this study, while those with PD were defined as non-responders (**Figure 3A**). The following subgroup analyses showed that both the PFS and OS of responders were significantly longer than those of non-responders (PFS: 266.0 *vs.* 52.0 days, *P*<0.0001; OS: 601.0 *vs.* 187.0 days, *P*=0.008) (**Figures 3B, C**).

**Figure 4:**
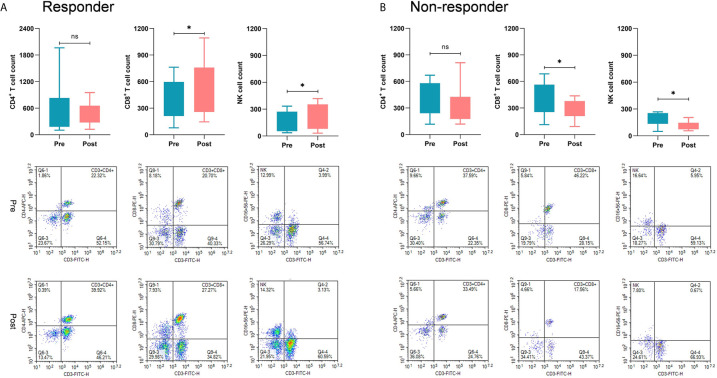
Comparisons of immunological response between responders and non-responders. **(A)** In the subgroup of responders, the counts of CD8^+^ T cells and NK cells were significantly increased after receiving immunoadjuvant infusion (*P* < 0.05). **(B)** In the subgroup of non-responders, the counts of CD8^+^ T cells and NK cells were markedly decreased after receiving immunoadjuvant infusion (*P* < 0.05). *p < 0.05; ns, not significant.

Moreover, we further investigated alterations in immune cell subsets in patients who received immunoadjuvant therapy. Twenty-four patients had PBMCs from peripheral blood samples available for immunological analysis. In the subgroup of responders, the counts of CD8^+^ T cells and NK cells were significantly increased after immunoadjuvant infusion (*P*<0.05). In contrast, the counts of CD8^+^ T cells and NK cells unexpectedly decreased in the subgroup of non-responders (*P*<0.05), while no obvious correlation was observed between treatment response and CD4^+^ T cell counts (**Figure 4**).

### Factors Associated With Treatment Response

We considered the prominent survival benefit of responders achieved after receiving immunoadjuvants and reirradiation and compared the baseline characteristics between responders and non-responders to explore potential factors associated with patients’ treatment response. The final results showed that responders had an older age at diagnosis (48.3 ± 10.6 *vs.* 37.8 ± 13.9 years, *P*=0.028) and a higher rate of local recurrence (53.3% *vs.* 13.3%, *P*=0.020) than non-responders (**Table 1**).

**Figure 5:**
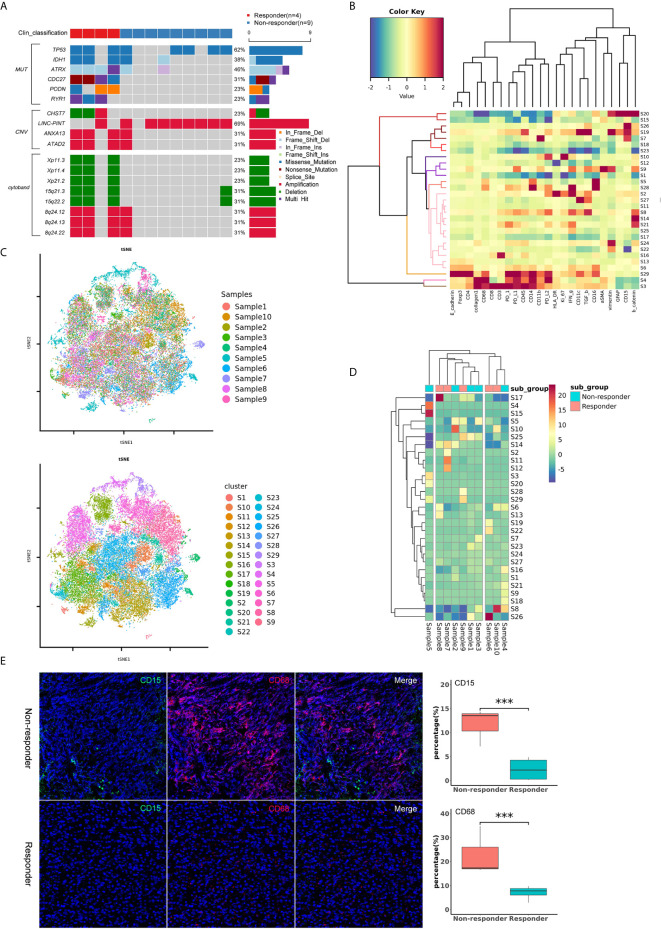
**(A)** Oncoplot of significantly different mutations, CNVs and cytobands between the responder and non-responder subgroups. **(B)** Heatmap showing the z-scored mean marker expression of the panel markers for each PhenoGraph cluster. Clusters and markers are grouped by expression profiles. **(C)** t-SNE plots of 43071 subsampled single cells from each PhenoGraph cluster identified in the heatmap image. Cells are colored by samples and clusters. **(D)** Heatmap showing the z-score of the mean percentage of single-cell clusters in each sample. Clusters and patients are grouped by the densities of single-cell clusters. **(E)** Imaging mass cytometry analysis of the tumor immune microenvironment between responders and non-responders. The non-responders showed higher percentages of CD15^+^ (green) and CD68^+^ (red) cells than the responders (*P* < 0.001). ***p value < 0.001.

WES was performed on 13 patients, including 4 responders and 9 non-responders. We then compared the frequency of the mutations between responders and non-responders. Apart from that of the known molecular marker IDH1, we found that the status of cell division cycle 27 (CDC27), podocon (PODN), α-thalassemia/mental retardation syndrome X-linked (ATRX) and ryanodine receptor type 1 (RYR1) was significantly different between the two subgroups (*P*<0.05) (**Figure 5A**). In particular, all four patients with CDC27 mutations responded, and the others without this mutation were confirmed as non-responders, reminding us of the significance of CDC27 in predicting the treatment response to immunotherapy. We also compared the frequency of CNVs and cytobands, and found several potential biomarkers, including carbohydrate sulfotransferase 7 (CHST7), 15q21.3 and 15q22.2, which were associated with treatment response (*P*<0.05) (**Figure 5A**).

Ten patients, including 5 responders and 5 non-responders, were successfully assessed with IMC. We identified 43071 single cells and quantified the expression of marker genes of each cell. Clustering with PhenoGraph identified 29 diverse cell phenotypes (**Figures 5B–D**). According to the comparison of cell clusters between responders and non-responders, we found that the percentage of CD15^+^ and CD68^+^ cells in the subgroup of non-responders was higher than that in the subgroup of responders (*P*<0.001, **Figure 5E**). These data suggest that CD15^+^ or CD68^+^ cells may play an important role in the tumor immune microenvironment of patients who receive immunotherapy.

### Survival Analysis

We then conducted univariate and multivariate survival analyses to better understand factors associated with patient prognosis. The univariate analysis confirmed treatment response (*P*<0.0001), age at diagnosis (*P*=0.007), and recurrence pattern (*P*=0.002) as prognostic factors for PFS, while treatment response (*P*=0.008), KPS score (*P*=0.033), and recurrence pattern (*P*=0.035) were confirmed as prognostic factors for OS (**Figures 3** and **S4**). The extent of resection showed potential for predicting survival, but it did not reach statistical significance (*P*=0.058 for PFS and *P*=0.051 for OS) (**Figure S4**). In the included Cox proportional hazard model, which shows all these prognostic factors screened by univariate analysis, treatment response was identified as an independent prognostic factor. The adjusted hazard ratio (HR) was 0.022 (95% CI: 0.004-0.126, *P*<0.001) for PFS and 0.323 (95% CI: 0.132-0.785, *P*=0.013) for OS.

## Discussion

Developments in the field of immunotherapy have recently provided new options for patients with GBM, especially when tumors progress after conventional treatments (30). However, to date, all phase III clinical trials of immunotherapy against GBM have reported unsuccessful results, largely blamed on tumor heterogeneity and an immunosuppressive microenvironment (6, 16). Previous studies suggested that radiation could prime the immune system to enhance the effciency of immunotherapy and that a combination of radiation with immunotherapy is more effective than monotherapy (18, 21, 23). In the present study, we evaluated the safety and efficacy of reirradiation plus immunoadjuvants in adult patients with recurrent WHO grade IV glioma and found that it was well tolerated and seemed to be effective. Patients who received this protocol achieved a median PFS of approximately three months and a median OS of approximately one year without any severe treatment-related adverse events, which appeared to be no significant survival advantage over previous salvage therapies (31). But of note, the median PFS and OS of responders was 266 and 601 days, respectively, which has been remarkably prolonged.

To our knowledge, this is the first study based on the combination of reirradiation and intracranial and systemic immunoadjuvants for recurrent malignant gliomas. It is believed that radiation can, to a certain extent, kill tumor cells and consequently result in the release of tumor neoantigens (18, 22). These antigens serve as “*in situ* vaccines” that can be recognized by the immune system and stimulate the infiltration of T cells. Reynders et al. systemically reviewed a rare clinical event: the abscopal effect, which was induced by radiation (23). The abscopal effect was defined as a phenomenon of tumor regression at nonirradiated, distant tumor sites (18, 23). In our study, we found that three (10.0%) patients had undergone the abscopal effect. For example, the tumors in the septum pellucidum, brainstem, and left temporal lobe of patient 2 simultaneously regressed when radiation was performed in the field of the septum pellucidum (**Figure S2**).

It should be noted that the success of immunotherapy depends on two simultaneous prerequisites: the availability of identifiable tumor antigens and adequate infiltration of effector T cells (32). Therefore, we introduced the application of intracranial and systemic immunoadjuvants concurrent with radiation. Poly I:C and GM-CSF are considered immunoadjuvants with high potential to boost immunological activity (14, 33), and have been widely used in the treatment of many tumors, including GBM (14, 34, 35). Traditionally, immunoadjuvants are given *via* systemic administration, such as intramuscular administration, which may only induce limited immunoactivity due to the existence of the BBB (36, 37). Thus, poly I:C was directly infused into the surgical cavity or ventricle in our study to achieve a maximal immunological response. Poly I:C is known to be a toll-like receptor 3 (TLR-3) agonist, which can facilitate maturation of dendritic cells (38). It has been reported poly I:C can prolong the survival of CD4+ T cells and enhance the proliferation of activated T cells, and that it is involved in the reactivation of tumor-infiltrating CD8+ T cells (39). Our results showed that poly I:C enhanced higher activation of CD8^+^ T and NK cells, but a less extent of CD4^+^ T cells in responders, which led to a more favorable CD8^+^:CD4^+^ T cell ratio. Notably, the increased counts of CD8^+^ T and NK cells were positively correlated with patient survival, which was in accordance with previous findings (40). In contrast, the counts of CD8^+^ T and NK cells in non-responders were decreased even after infusion of immunoadjuvants, which implied that the immune cells had already been exhausted thereby indicating a poor outcome in these patients.

With respect to the safety profile for immunoadjuvants, we found that the most common AEs were fever, vomiting, headache, and fatigue. Fortunately, no severe AEs have been observed, consistent with the results reported in other clinical trials (36, 37). All these data suggested that our regimen was safe and well-tolerated. Aside from the safety and efficacy of this regimen, identifying the subgroup of patients who would obtain clinical benefits is also of great significance. Therefore, in this study, we systematically explored the characteristics of responders and non-responders. The final results demonstrated that the responders had an older age at diagnosis and a lower rate of distant recurrence. Generally, older patients tended to show a relatively worse treatment response than younger patients because of decreased immune system effectiveness (41, 42). In this study, non-responders seemed to be more common in the younger patient group, which could be partly explained by the different frequencies of H3K27M-mutant DMG between non-responders and responders (20.0% *vs.* 13.3%). As we all know, H3K27M-mutant DMG is a malignancy predominately found in children and young adults and is concurrent with a poor immune response (43). Distant recurrence is regarded as a sign of late stage disease in patients whose immune systems have declined and tumoral immune escape has enhanced (44). Hence, patients with local recurrence are more likely to exhibit a favorable immune response.

In addition, our results showed that CDC27, PODN, ATRX and RYR1 status was significantly different between responders and non-responders. In particular, CDC27, a gene correlated with tumor progression and programmed death ligand-1 expression (45), was mutated in all responders and wild-type in all non-responders, which indicated great significance in predicting the treatment response of immunotherapy. We also found that CHST7, 15q21.3 and 15q22.2 could serve as potential biomarkers predicted for treatment response. Furthermore, by analyzing the features of the tumor immune microenvironment of patients, we found that the percentages of CD15^+^ and CD68^+^ cells in non-responders were higher than those in responders. It has been reported that both CD15 and CD68 are immunosuppressive markers that play an important role in suppressing T-cell-mediated immunity (46, 47). CD68 is a major biomarker for the quantification of tumor-associated macrophages (TAMs) (48). As we all know, TAMs are a well-recognized core element of tumor microenvironment (TME) and generally characterized as M2-like macrophages which are associated with tumor progression and poor prognosis (49, 50). This is the reason that high tumor CD15 and CD68 expression indicates a limited response and poor survival. Therefore, all these biomarkers might be exploited as potential therapeutic targets for malignant gliomas in the future.

Limitations do exist in our study. First, it is a study from a single institution, which to some extent decreases the stability of the conclusion. Second, the potential molecular mechanism of patients who responded to this treatment protocol has yet to be clarified. We collected the cerebral spinal fluid of patients before, during, and after treatment and made some interesting observations. We believe the mechanism can be elucidated in the near future. Third, the amount of Tregs has not been detected in our research, which was important in assessing the effect of CTX in ablating Tregs. Finally, we should continue this study until the last patient has reached the endpoint because there are still 7 patients alive at the current stage.

## Conclusions

In summary, this trial demonstrated that the combination of immunotherapy and radiotherapy was well tolerated. Low-dose reirradiation plus intracranial and systemic immunoadjuvants has shown promising immunological responses and clinical benefits in patients with recurrent WHO grade IV gliomas. These data support a larger phase II study of this regimen in patients with recurrent GBM, in which feasibility will be assessed in multicenter settings and efficacy will be evaluated in comparison with that of controls.

## Data Availability Statement

The original contributions presented in the study are included in the article/**Supplementary Material**. Further inquiries can be directed to the corresponding author.

## Ethics Statement

The studies involving human participants were reviewed and approved by Institutional Review Board of Capital Medical University. The patients/participants provided their written informed consent to participate in this study.

## Author Contributions

Acquisition of data: HJ, ML, CY, and XZ. Analysis and interpretation of data: HJ, KY, YC, and XR. Statistical analysis: HJ and KY. Drafting of the article: HJ and SL. Funding acquisition: SL and YC. Conception and design: YC and SL and study supervision: SL. All authors contributed to the article and approved the submitted version.

## Funding

This work was supported by the National Natural Science Foundation of China (81771309) and the Capital’s Funds for Health Improvement and Research (2020-2-1075).

## Conflict of Interest

The authors declare that the research was conducted in the absence of any commercial or financial relationships that could be construed as a potential conflict of interest.

## Supplementary Material

Supplementary Figure 1 Representative images of patients with local and distant recurrence.

Supplementary Figure 2 The pre- and posttreatment MR images of patient 2. The patient experienced both local **(A)** and distant **(B, C)** recurrence. After one cycle of treatment, the lesion in the left temporal lobe **(E)** and metastases in the septum pellucidum **(F)** and brainstem **(G)** were in remission. But a new lesion was found in the left cerebellum **(D, H)**. Therefore, the treatment response of this patient was defined as PD.

Supplementary Figure 3 The pre- and posttreatment MR images of patient 22. The patient had a recurrent lesion in the midbrain **(A)**. After one cycle of treatment, the lesion disappeared **(C)**. However, new lesions occurred in the bilateral thalamus and basal ganglia **(B, D)**. Therefore, the treatment response of this patient was defined as PD.

Supplementary Figure 4 Univariate survival analyses of the prognostic factors.
