# Supplementary figures and images for: Combination of Immunotherapy and Radiotherapy for Recurrent Malignant Gliomas: Results From a Prospective Study
Source: Front Immunol. 2021 May 7;12:632547. doi: 10.3389/fimmu.2021.632547 (PMC8138184; doi:10.3389/fimmu.2021.632547)

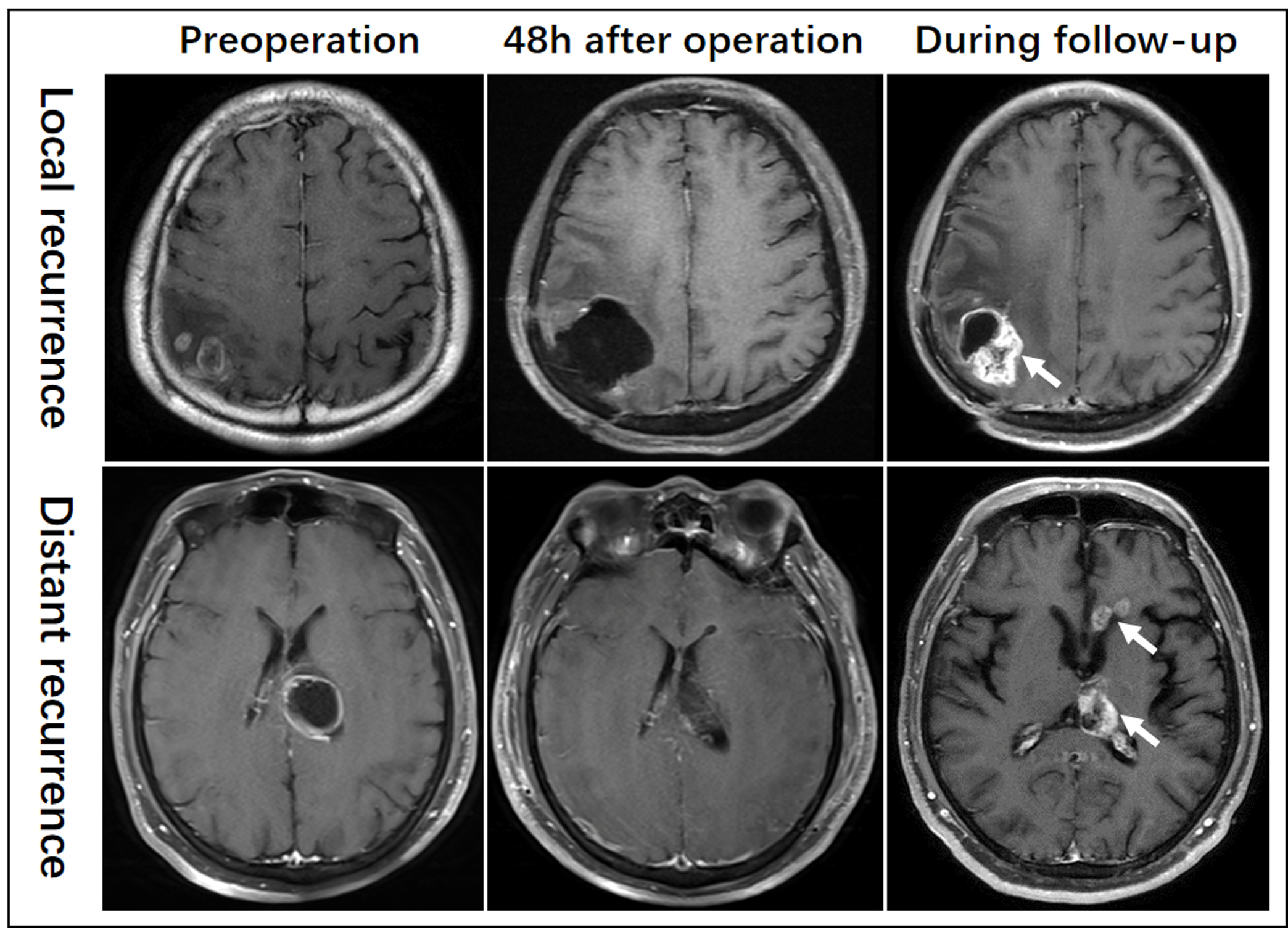

Supplement: Supplementary Figure 1 — Representative images of patients with local and distant recurrence. [file Image_1.jpeg]

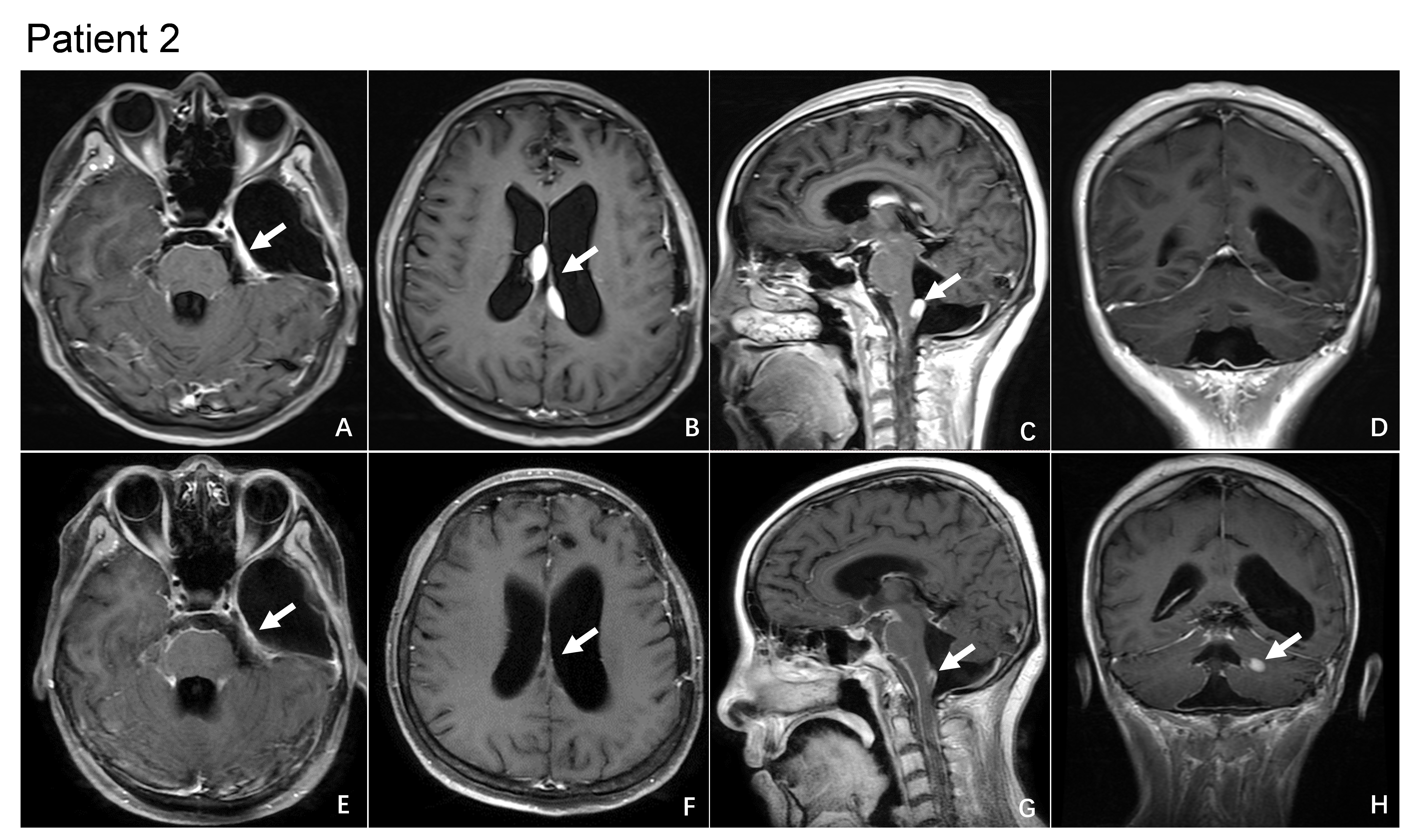

Supplement: Supplementary Figure 2 — The pre- and posttreatment MR images of patient 2. The patient experienced both local (A) and distant (B, C) recurrence. After one cycle of treatment, the lesion in the left temporal lobe (E) and metastases in the septum pellucidum (F) and brainstem (G) were in remission. But a new lesion was found in the left cerebellum (D, H). Therefore, the treatment response of this patient was defined as PD. [file Image_2.jpeg]

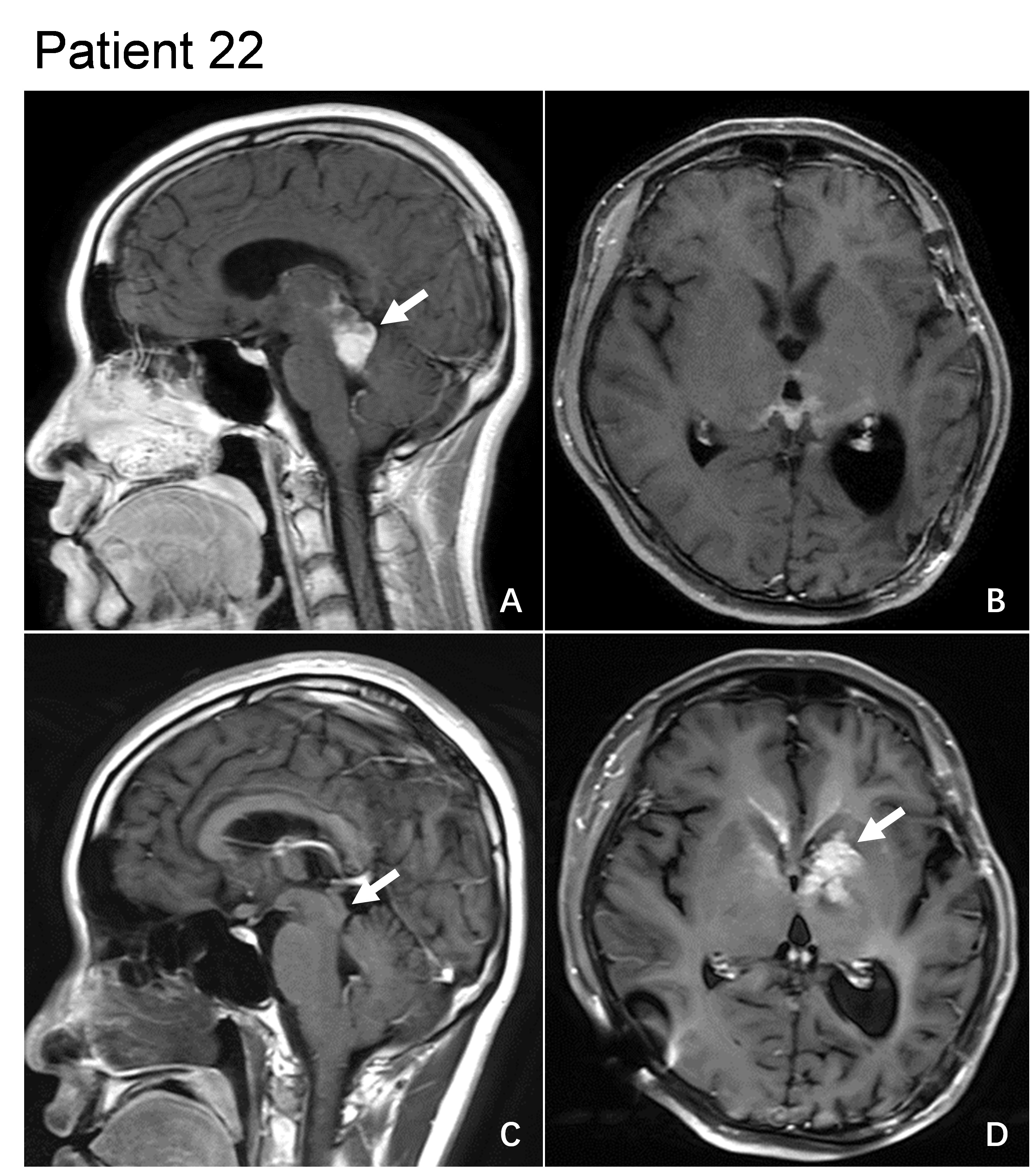

Supplement: Supplementary Figure 3 — The pre- and posttreatment MR images of patient 22. The patient had a recurrent lesion in the midbrain (A). After one cycle of treatment, the lesion disappeared (C). However, new lesions occurred in the bilateral thalamus and basal ganglia (B, D). Therefore, the treatment response of this patient was defined as PD. [file Image_3.jpeg]

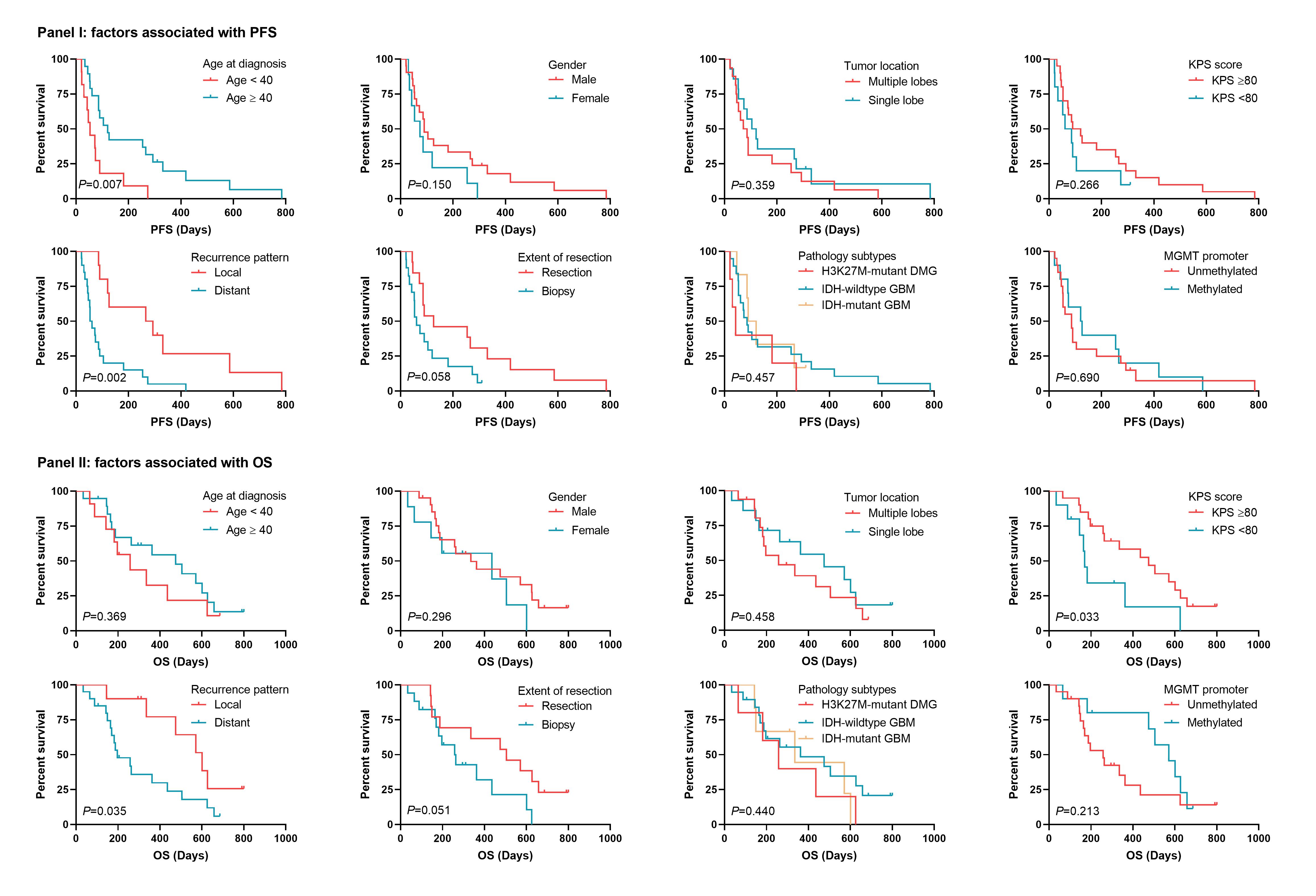

Supplement: Supplementary Figure 4 — Univariate survival analyses of the prognostic factors. [file Image_4.jpeg]
