# Supplementary material for: Combination of Immunotherapy and Radiotherapy for Recurrent Malignant Gliomas: Results From a Prospective Study
Source: Front Immunol. 2021 May 7;12:632547. doi: 10.3389/fimmu.2021.632547 (PMC8138184; doi:10.3389/fimmu.2021.632547)
Supplement: Supplementary file 5 [file Table_1.doc]

TABLE S1 List of antibodies and corresponding metal isotope labels used in study.

| **No.** | **Label** | **Antibody** | **Vendor** | **Catalog** | **Clone** | **Dilution** |
| --- | --- | --- | --- | --- | --- | --- |
| 1 | Er166 | CD45 | Biolegend | 368502 | 2D1 | 50 |
| 2 | Er170 | CD3 | Fluidigm | 3170019D | Polyclonal, C-Termina | 50 |
| 3 | Gd156 | CD4 | Fluidigm | 3156033D | EPR6855 | 50 |
| 4 | Gd155 | Foxp3 | Biolegend | 320102 | 206D | 50 |
| 5 | Dy162 | CD8a | Fluidigm | 3162035D | D8A8Y | 50 |
| 6 | Sm149 | CD11b/Mac-1 | Fluidigm | 3149028D | EPR1344 | 50 |
| 7 | Sm154 | CD11c | Fluidigm | 3154025D | Polyclonal | 50 |
| 8 | Nd144 | CD14 | Fluidigm | 3144025D | EPR3653 | 50 |
| 9 | Eu153 | CD15 | Biolegend | 301902 | HI98 | 50 |
| 10 | Nd146 | CD16 | Fluidigm | 3146020D | EPR16784 | 50 |
| 11 | Nd142 | CD19 | Fluidigm | 3142014D | 6OMP31 | 50 |
| 12 | Nd145 | CD33 | Fluidigm | 3145017D | Polyclonal | 50 |
| 13 | Sm152 | CD56 | Abcam | 9018 | RNL-1 | 50 |
| 14 | Tb159 | CD68 | Biolegend | 916104 | KP1 | 50 |
| 15 | Sm147 | IFN-γ | Abcam | 9657 | Polyclonal | 50 |
| 16 | Yb174 | HLA-DR | Fluidigm | 3174023D | YE2/36 HLK | 50 |
| 17 | Nd150 | CD274/PD-L1 | Fluidigm | 3150031D | E1L3N | 50 |
| 18 | Yb172 | CD273/PD-L2 | Fluidigm | 3172028D | 176611 | 50 |
| 19 | Ho165 | CD279/PD-1 | Fluidigm | 3165039D | EPR4877(2) | 50 |
| 20 | Pr141 | α-SMA | ebioscience | 14-9760-82 | 1A4 | 200 |
| 21 | Tm169 | collagen I | Abcam | 88147 | 3G3 | 100 |
| 22 | Gd158 | CD324/E-Cadherin | Abcam | 231303 | 4A2 | 50 |
| 23 | Nd148 | Vimentin | Abcam | 8978 | RV202 | 200 |
| 24 | Er168 | Ki-67 | Biolegend | 350502 | Ki-67 | 50 |
| 25 | Lu175 | b-catenin | Biolegend | 844603 | 12F7 | 50 |
| 26 | Yb176 | GFAP | Abcam | 223127 | EPR19996 | 200 |
| 27 | Eu151 | TGF-β | Biolegend | 846802 | O92B5 | 50 |
